# Musical Rounds: A multisite, mixed-methods feasibility study of a musical legacy session in palliative care

**DOI:** 10.1017/S1478951526102624

**Published:** 2026-05-07

**Authors:** Melanie Ambler, Mukta Awasthi, Lynn Gerber, Bryant Lin, Claire E Wakefield

**Affiliations:** 1 School of Medicine, Stanford University, Stanford, CA, USA; 2 Department of Medicine, VA Palo Alto Healthcare System, Palo Alto, CA, USA; 3 Department of Anesthesiology, Santa Clara Valley Medical Center, San Jose, CA, USA; 4 Division of Primary Care and Population Health, School of Medicine, Stanford University, Palo Alto, CA, USA; 5 Behavioural Sciences Unit, School of Clinical Medicine, UNSW Medicine and Health, Discipline of Paediatrics and Child Health, UNSW Sydney, Kensington, NSW, Australia; 6 Division of Quality of Life and Pediatric Palliative Care, Department of Pediatrics, Stanford University and Stanford Medicine Children’s Health, Palo Alto, CA, USA

**Keywords:** Music, palliative care, critical care, pain, anxiety

## Abstract

**Objectives:**

Patients hospitalized with a life-limiting illness, along with their loved ones, frequently experience anxiety, stress, and pain. Legacy building through storytelling and music may alleviate emotional strain and provide comfort. Musical Rounds is a novel music medicine program designed to reduce distress and support legacy building for adult patients receiving palliative care and their loved ones.

**Methods:**

This multisite, mixed-methods, pre–post feasibility study was conducted across 3 hospitals in California, USA. Participants engaged in live bedside recording sessions in which personal stories were shared with real-time musical improvisation provided by a clinician-musician. Afterward, participants received a personalized edited recording combining voice and improvised music. Pain, stress, anxiety, and comfort were assessed before and after each session using a 0–10 numeric rating scale. Perceived mood changes were assessed through directed qualitative content analysis.

**Results:**

We invited 100 adult patients hospitalized with a life-limiting illness and their loved ones to participate. If patients were unable to respond, loved ones participated on their behalf. Patients (*n* = 79) demonstrated statistically significant within-group differences between pre- and post-session assessments, including lower pain (−1.58, *p* < .001), stress (−2.89, *p* < .001), and anxiety (−2.73, *p* < .001), and higher comfort (+1.61, *p* < .001). Loved ones (*n* = 42) reported lower stress (−3.14, *p* < .001) and anxiety (−2.86, *p* < .001), and higher comfort (+1.83, *p* = .004). Directed content analysis indicated perceived mood improvement in 59% (47/80) of patients and 68% (30/44) of loved ones.

**Significance of results:**

Musical Rounds, a personalized music and storytelling session for hospitalized patients with life-limiting illness and their loved ones, was associated with lower self-reported stress, pain (patients only), and anxiety, and higher comfort and perceived mood across 3 hospitals. Findings demonstrate the feasibility and suggest potential benefits of music medicine–supported legacy building in palliative care. Controlled studies with independent assessors are needed to further evaluate efficacy.

## Introduction

The field of palliative care aims to enhance quality of life (Rantanen et al. 2022) and address underlying dignity-related distress (Chochinov et al. 2009), while recognizing that the experience of hospitalization with a life-limiting illness affects not only the patient but also their loved ones. Dignity therapy aims to address this distress by encouraging patients with life-limiting diagnoses to reflect on their lives during recorded sessions (Chochinov et al. 2005; Kittelson et al. 2019). This practice has been shown to be effective in improving hope, quality of life, anxiety, and depression (Zhang et al. 2022; Zheng et al. 2023).

In addition to dignity therapy, other methods of building legacy include music-based interventions. The “Song of Life” intervention involves patients participating in a music therapy intervention that creates a biographically meaningful song (Warth et al. 2021). Participants in the music therapy group reported higher spiritual well-being and ego-integrity, lower distress, and satisfaction compared to a relaxation control (Koehler et al. 2022). Music preference has been shown to be an important factor in modulating pain relief (Cepeda et al., n.d.; Van der Valk Bouman et al. 2024).

An estimated 10,000 music therapists currently work in the United States, with only 42% of those music therapists working in the adult population, and an even smaller portion working inpatient (American Music Therapy Association 2021 Workforce Analysis 2021). Given that over 34 million patients were admitted to hospitals nationwide in 2025, with the overwhelming majority of those being adults (approximately 84%), access to music therapy is unfortunately limited for most adults hospitalized in the United States (AHA Hospital Statistics: Fast Facts on U.S. Hospitals 2025 2025). However, the emerging field of music medicine helps to broaden the spectrum of musical offerings available to patients, as it can be provided by clinically trained professional musicians (MHTP 2024; Power of Music in Healthcare | Peabody Institute n.d.) or healthcare clinicians who are musicians (Orchestra Directory n.d.; Wong 2014) in the absence of a music therapist.

Music medicine does not replace the field of music therapy but rather widens access to music in the healthcare setting, bolstering the vital role that music plays in addressing well-being (Hole et al. 2015; Kühlmann et al. 2018; Thompson et al. 2021). One recent study compared music medicine with music therapy in patients with cancer, finding that both were effective in enhancing target outcomes measured using quantitative assessments (e.g. via visual analogue scale and numeric rating scale [NRS]), with qualitative data suggesting that music therapy was preferred by patients (Bradt et al. 2015).

To date, limited studies have evaluated the impact of personalized music medicine for patients receiving palliative care, in whom rates of pain, stress, anxiety, and discomfort are often high (Carlson 2016; Satsangi and Brugnoli 2018; Inoue et al. 2024). One scoping review identified 6 potential areas of benefit that emerged from the existing literature in music and palliative care: pain management, relaxation, happiness/hope, anxiety and depression management, enhanced spirituality, and improved quality of life (Nyashanu et al. 2021).

There are few studies that currently address music medicine for supporting loved ones, although evidence suggests their inclusion in patient care can decrease rates of stress and anxiety, especially in critical or palliative care (Amass et al. 2020; Love Rhoads et al. 2022; Shirasaki et al. 2024).

To address the issues raised above and in collaboration with palliative care physicians, music therapists, medical psychologists, and professional musicians, we developed a novel music medicine intervention entitled “Musical Rounds.” Musical Rounds is a bedside recording session offered by a trained musician-clinician that includes personal storytelling and musical improvisation. A legacy recording of the participant’s voice with an accompanying musical soundtrack is produced after each session and returned to the participant. This study aimed to explore the potential impact of Musical Rounds on overall well-being by assessing pain, stress, anxiety, comfort, and mood in patients and their loved ones before and after participation in the intervention.

## Methods

### Study design

This was a multicenter, mixed-methods, pre–post intervention study (see STROBE checklist in Supplementary File 1).

### Setting

Patients and loved ones were recruited at Stanford Health Care (SHC), Santa Clara Valley Medical Center (SCVMC), and the Veterans Affairs Palo Alto (VAPA) after Institutional Review Board approval was received at each site (SHC: 75283, approved 7/1/2024; SCVMC: 24-019, approved 8/20/2024; VAPA: LEL0001, approved 2/26/2025). These 3 sites represent an academic institution (SHC), a county hospital (SCVMC), and the VA (VAPA), each of which serves varied populations.

### Participants

Patients were invited to participate if they were: (a) referred by the palliative care teams at SHC, SCVMC, or VAPA or the social workers in the intensive care unit (ICU) at SCVMC (due to small size of palliative care); (b) ≥18 years of age; (c) spoke English or another language covered by the hospital interpreter service (i.e. Spanish, Mandarin, Vietnamese, etc.); and (d) were able to provide informed written consent. Loved ones were invited to participate if they met the inclusion criteria (b) through (d) above. If the patient was unresponsive or unable to participate, the loved one could choose to participate themselves and provide consent for the patient, acting as their legally authorized representative. Loved ones included parents, partners, siblings, or other legally authorized representatives. If a loved one participated on behalf of a patient, they shared their personal stories about their loved one. Patients were ineligible if they were: (a) on isolation precautions or (b) unable to verbally participate in the interview session and did not have a loved one present to consent and participate on their behalf.

### Recruitment

The musician-clinician or care team approached patients at the bedside between August 2024 (for SHC and SCVMC, March 2025 for VAPA) and May 2025 to assess initial interest. If the individual(s) expressed interest, the musician-clinician informed the participant of the study goals, risks, and benefits, and participants provided written informed consent for inclusion. Musical Rounds was conducted by a musician-clinician (a professional cellist and final year medical student). As such, participants were informed that this session was not music therapy. The musician-clinician, however, was trained to offer Musical Rounds and contributed significantly to its development (Musical Rounds training and facilitator guide available upon request).

Palliative care team members were invited to be present for any session. Participants were given the option to allow Musical Rounds to share their recording with the public (e.g. in podcast form), but this was not required for inclusion. If the individual(s) verbally declined participation in the study altogether, the reason was collected with no identifying information. Participation in Musical Rounds was available only through enrollment in the research study.

### Musical Rounds session

**Figure 1.**
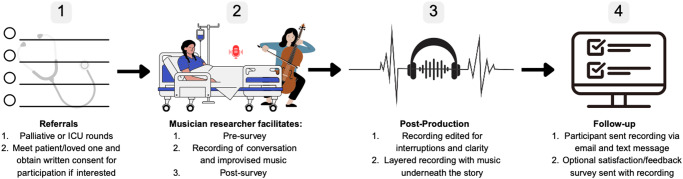
Musical Rounds protocol. Figure 1 long description.

We invited participant(s) to work with the musician-clinician during a recording session that took place at the patient’s bedside in a private or shared room (see Figure 1).

There were 3 options for participation: 1) patient alone; 2) dyad of patient and loved one; and 3) loved one alone if the patient was unable to communicate.

Following recruitment, the musician-clinician guided the patient (and/or loved one) through a verbal pre-intervention survey. Study data were collected and managed using REDCap electronic data capture tools hosted at Stanford University (Harris et al. 2009, 2019). Participants were asked to rate their current pain, stress, anxiety, and comfort levels using an NRS from 0 to 10 (Krebs et al. 2007; Prokopowicz et al. 2022; LiaBraaten et al. 2023). Participants were also asked open-ended questions about their relationship with music, why they chose to participate, their mood, and musical preferences. The questions were determined by the multidisciplinary study team.

After the pre-session survey, the musician set up a portable recording device (Zoom e6 audio recorder; 2 lavalier microphones, 1 for patient and 1 for loved one; a Neumann MCM 114 microphone for the cello). They then started the recording and facilitated a conversation with the participant about memories from their life, providing prompts such as “transport me to your favorite place on Earth” *or* “tell me about your relationship with ____” (see Supplementary Recording 2).

While the participant responded, the musician-clinician considered a musical improvisation that could represent the story shared by the participant. She then composed this piece of music on the spot and performed it in real time for the participant following a response to one of the prompts. The session typically contained 3 stories and subsequent musical improvisations. The musician-clinician checked in after each musical piece to see if the participant wanted to continue. If a session ran to 45 minutes in duration, the musician concluded the session at the next appropriate juncture.

After the recording session, the musician-clinician again repeated a survey of pain, stress, anxiety, comfort, and mood. Participants were asked for feedback, both positive and negative. The musician clarified whether the participant was willing for their recording to be shared with the public or whether they preferred it be kept private.

The recording was edited for interruptions and clarity by the musician-clinician using Logic Pro software (Logic Pro for Mac n.d.). Within 3 business days of the recording session, the participant received 2 electronic versions of the edited recording: one containing the participant’s voice with the newly composed music as a layered soundtrack and the other containing only the music. Participants were asked to confirm receipt of their recording and given an optional feedback survey to complete, in which they were asked for their satisfaction with Musical Rounds (very satisfied, satisfied, neutral, dissatisfied, very dissatisfied), open-ended feedback, and why/why not they wanted their recording shared. If participants did not respond, we contacted the participant a maximum of 2 further occasions via text message, phone call, or email.

We conducted a retrospective medical chart review with a sample of the first 34 patients at SHC to compare patients’ pain level as reported to nursing staff prior to the session and compared this to the pain level reported to the musician-clinician prior to the session start. These data were pulled directly from the “pain” tab in the Epic medical record system, with the most recent pain measurement prior to the session recorded (within 2 hours of the session). We performed a Spearman correlation to assess the reliability of patients’ pain level reported to nursing staff as compared to that reported to the musician-clinician.

To support future replicability of the program, we developed a facilitator guide and standardized training protocol for replicating Musical Rounds at other sites. These are available upon request.

### Statistical analyses

Assuming a small-to-moderate effect size and a bivariate normal population with *r* = 0.30, a required sample size of *n* = 85 was calculated based on a statistical power of 0.80 and a Type I error rate of *α* = 0.05 (Cohen 2013). We employed a Bonferroni correction due to the multiple hypotheses (pain, stress, anxiety, and comfort) being tested, with an adjusted significance level of 0.0125.

Hypotheses for the primary pre–post outcomes of pain, stress, anxiety, and comfort were analyzed using R (R Core Team 2020; Team RC 2013). We compared complete pre–post data using the Wilcoxon signed-rank paired test, as well as a paired permutation test (*n* = 1000), with the greatest *p*-value between the 2 tests reported. Missing data were excluded from analysis.

### Qualitative analysis

We performed directed content analysis on the qualitative data collected to assess for changes in self-reported mood before and after the Musical Rounds session (Hsieh and Shannon 2005; Assarroudi et al. 2018). Two physicians trained in directed content analysis graded pre–post mood responses for 1) improvement, 2) no change, or 3) worsening. The reviewers then compared responses, and in the case of disagreement, discussed their analyses. If the reviewers were unable to reach a consensus, a third clinician was asked to provide their grade for the set of responses.

## Results

**Figure 2.**
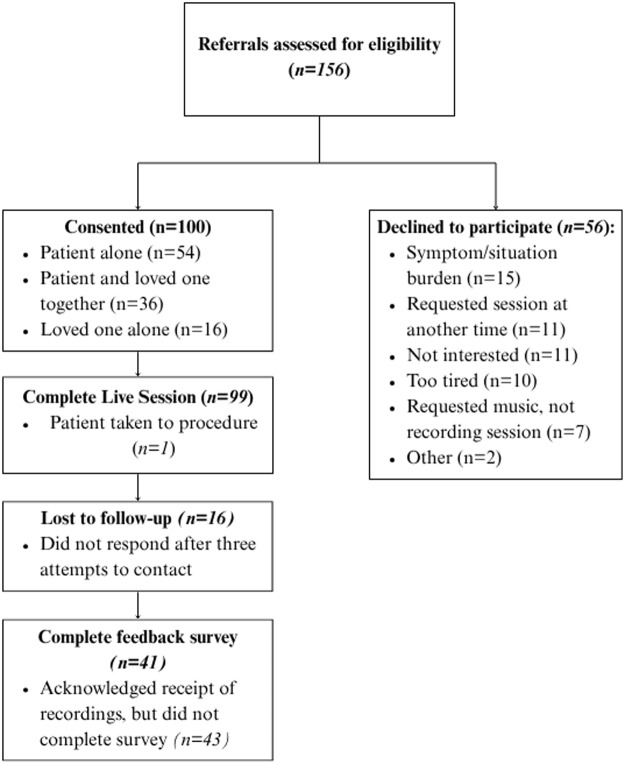
Recruitment diagram. Figure 2 long description.

Musical Rounds sessions were conducted between August 2024 and May 2025. Of 156 referrals, 100 sessions were conducted, and 56 were declined (Figure 2). Of the 100 sessions performed, 51% (*n* = 51) were with the patient alone, 32% (*n* = 32) were with the patient and loved one together, and 17% (*n* = 17) were with the loved one alone. One session was interrupted, resulting in 99 complete live sessions. The mean recording session length was 25.7 minutes (standard deviation [SD] = 7.57), and mean edited recording length was 16.4 minutes (SD = 6.13). Complete pre–post data were collected for 95% of patients (*n* = 79/83) and for 86% of loved ones (*n* = 42/49). On follow-up, 84% (*n* = 84) confirmed receipt of recordings, with 41% (*n* = 41) completing the optional feedback survey and 43% (*n* = 43) confirming receipt via email or text. A small proportion of individuals (*n* = 16) were lost to follow-up after 3 attempts to contact.

Patients’ mean age was 63.3 years (SD = 17.4, range 20–102 years). The most frequent diagnosis was cancer (70/100 patients). Four sessions were conducted in languages other than English: Spanish (*n* = 2), Korean (*n* = 1), or Vietnamese (*n* = 1). Median length of stay prior to a session was 10 days (range: 1–133 days). The mean age of loved ones was 53.7 years (SD = 17.4), with spouse being the most common relationship (*n* = 19, 40%). Participants provided consent for public sharing in 74 sessions (74%). Further demographic and clinical characteristics are provided in Table 1.

**Table 1.** Characteristics of consented participants Table 1 long description.

Descriptive demographics			
Characteristic	Patient (*n* = 100)		Family (*n* = 48)
Participants per site (%, *n*)		Gender (female:male)	31:17
SHC	59% (59)	Age (mean, SD, years)	53.72 (17.39)
SCVMC	32% (32)	Relationship to patient	
PAVA	9% (9)	Spouse	40% (19)
Age (mean, SD, years)	63.31 (17.38)	Child	29% (14)
Gender (female:male)	57:43	Parent	15% (7)
Race (%, *n*)		Friend	8% (4)
White	44% (44)	Grandchild	6% (3)
Asian	23% (23)	Other	6% (3)
Other	28% (28)		
Black/African American	5% (5)		
Ethnicity (%, *n*)			
Non-Hispanic	72% (72)		
Hispanic/Latino	27% (27)		
Declined	1% (1)		
Median length of stay prior to session (median, range, days)	10 (1–133)		
Language (%, *n*)			
English	96% (96)		
Spanish	2% (2)		
Korean or Vietnamese	2% (2)		
Diagnosis (%, *n*)			
Oncology	70% (70)		
Neurological	7% (7)		
Cardiac	8% (8)		
Respiratory failure	6% (6)		
Other	9% (9)		
Team referral (%, *n*)			
Palliative care	89% (89)		
Intensive care unit	11% (11)		
Session type (%, *n*)			
Patient alone	51% (51)		
Loved one alone	17% (17)		
Patient + loved one	32% (32)		
Consent to public sharing of recording	74% (74)		
Recording length (mean, SD, minutes)			
Raw session	25.7 (7.57)		
Edited and layered	16.4 (6.13)		

SHC = Stanford Hospital Center, SCVMC = Santa Clara Valley Medical Center, PAVA = Palo Alto Veterans Affairs. Race and ethnicity were extracted from the patient’s medical record; loved ones were not asked to report race or ethnicity.

### Patient-reported outcomes

**Figure 3.**
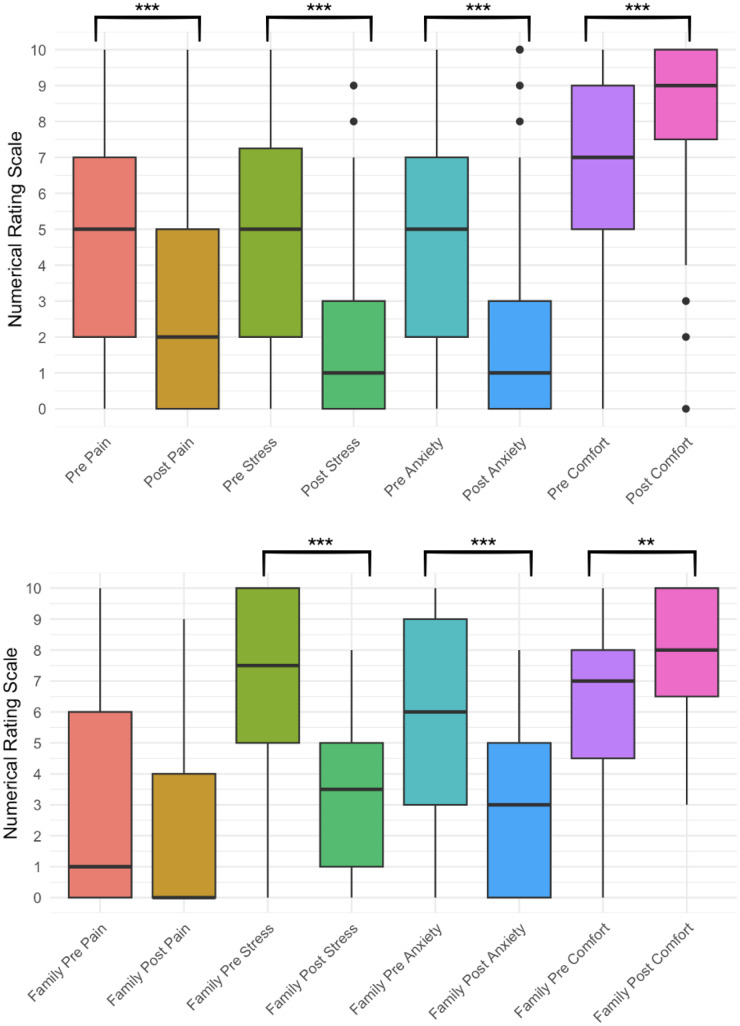
Boxplots of pre- and post-numeric rating scale outcomes: boxplots with median denoted by solid line. Asterisks denote results of pre–post comparison analysis using Wilcoxon signed-rank test and permutation test. ***p < .001, **p < .01. Figure 3 long description.

Patients’ self-reported data were associated with significant within-group differences in pain, stress, anxiety, and comfort levels following a Musical Rounds session (see Table 2, Figure 3).

**Table 2.** Pre–post numeric rating scale outcomes Table 2 long description.

Differences in numeric rating scale pain, stress, anxiety, and comfort pre- and post-session									
	Patient					Loved one			
	*N*	*M*	SD	*p*-value		*N*	*M*	SD	*p*-value
Pain					Pain				
Baseline	79	4.35	2.91		Baseline	35	2.80	3.59	
Post-intervention	79	2.77	2.79		Post-intervention	35	2.06	2.80	
Difference		**−1.58**		**<.001**	Difference		−0.74		.352
Stress					Stress				
Baseline	80	4.83	3.67		Baseline	42	6.73	3.04	
Post-intervention	80	1.94	2.40		Post-intervention	42	3.60	2.63	
Difference		**−2.89**		**<.001**	Difference		**−3.14**		**<.001**
Anxiety					Anxiety				
Baseline	80	4.65	3.32		Baseline	42	5.95	3.16	
Post-intervention	80	1.93	2.61		Post-intervention	42	3.10	2.58	
Difference		**−2.73**		**<.001**	Difference		**−2.86**		**<.001**
Comfort					Comfort				
Baseline	80	6.81	2.57		Baseline	35	6.26	2.93	
Post-intervention	79	8.41	2.14		Post-intervention	35	8.09	2.01	
Difference		**1.61**		**<.001**	Difference		**1.83**		**.004**

*N* = sample size, *M* = mean, SD = standard deviation. Reported *p*-values are taken from Wilcoxon signed-rank test and permutation test. Of note, the outcomes of pain, comfort, and music in healthcare questions were added to the family survey after recruitment had begun. This explains the discrepancy in *N* for those questions. The greatest *p*-value for each outcome measure is reported in the table. Bolded values denote statistical significance.

Self-reported pain scores were on average 36% lower after the session, declining from 4.35 to 2.77 (*p* < .001). One patient shared, “It made me not feel the pain. Music carried me on until I got my pain meds.” Another patient reported, “I didn’t feel any pain while she was playing” with their loved one, responding that “getting her to say she had no pain and she forgot all about it, that’s a miracle in and of itself.” For the subgroup of 34 patients included in the medical chart review, a positive correlation (*r* = 0.706) was observed between patient-reported pain recorded by the nursing team before the recording session (mean = 4.18, SD = 2.76) and that reported to the musician-clinician (mean = 4.35, SD = 2.77).

Self-reported stress levels were 60% lower post-session, decreasing from 4.83 to 1.94 (*p* < .001), and self-reported anxiety levels were 59% lower, from 4.65 to 1.93 (*p* < .001). One patient shared that their “stress definitely came down. Increased relaxation. Feeling this session went really good. I’m in the hospital, so my mood can be up and down like my stress. For this, I was just in a whole different space.”

Self-reported comfort scores were 24% higher after the session, increasing from 6.81 to 8.41 (*p* < .001). A patient reflected, “you made me feel comfortable; I don’t usually feel this able to share.”

Some sessions resulted in a silence of over 2 minutes after the music was played, with one patient reporting that the music “helped us bring to the surface buried thoughts and feelings,” and another reflecting that “there is something very healing about sharing experiences through music. It’s kind of like reverse storytelling. You’re the one telling the story, but it comes from me.”

### Loved ones’ self-reported outcomes

Loved ones’ self-reported data indicated significant within-group differences in stress, anxiety, and comfort levels before and after the Musical Rounds session (see Table 2, Figure 3).

Mean self-reported pain scores were not associated with significant differences before and after the session (2.80–2.06; *p* = .352). Mean stress scores were 47% lower post-session (6.73–3.60, *p* < .001), and mean anxiety scores were 48% lower (5.95–3.10, *p* < .001). In the follow-up survey, one spouse reported, “My anxiety, stress, and the feeling of being pressured went down gradually until the time I went to bed. I was so relaxed and had a sound sleep that night.”

Mean comfort scores were 29% higher after the session (6.26–8.09, *p* = .004). One participant reflected, “You’re giving me comfort, and I need to talk I think because I’m keeping it inside.”

### Perceived impacts on mood: Directed content analysis of qualitative data

For patients, 59% (*n* = 47) reported an improvement in their mood (36% no change, 5% worsening). For loved ones, 68% (*n* = 30) reported an improvement in their mood (30% no change, 2% worsening). Initial reviewer agreement was 83.8% (67/80) for patients and 90.9% (40/44) for loved ones, and reached 100% consensus after discussion, with no need for a third reviewer to reach consensus.

### Worsening of outcomes

Of note, there were instances in which patients or loved ones reported worsening in their pain (*n* = 9; *n* = 3, respectively), stress (*n* = 5; *n* = 2), anxiety (*n* = 6; *n* = 2), comfort (*n* = 5; *n* = 3), or mood (*n* = 4; *n* = 1). One reflected, “I’m feeling more emotional, I just don’t want them to deal with being without me,” and another said of disease, “It’s very unfair. S*** like this should not happen to good people.” If a session was particularly emotional, the palliative care team was notified for debriefing with the participant(s).

### Satisfaction (follow-up survey)

Of the 37 patients who responded to the optional follow-up survey, 84% (*n* = 31) reported being very satisfied, 14% (*n* = 5) were satisfied, and 2% (*n* = 1) reported feeling neutral regarding their experience with Musical Rounds. Of the 36 loved ones who responded, 94% (*n* = 34) were very satisfied, and 6% (*n* = 2) were satisfied. After the death of her husband, one participant said, “I cannot tell you how much your recording and music means to me and our girls. I listen to it every night … It is really just so beautiful, and to have his voice and laughter captured for all time, thank you!” (see supplementary audio file).

## Discussion

Musical Rounds, a personalized music-and-storytelling session for hospitalized patients with life-limiting illness and their loved ones, was associated with lower reported stress, pain (patients only), and anxiety, alongside higher comfort and perceived mood across 3 hospitals. These findings suggest that Musical Rounds is a feasible novel approach for legacy building in palliative care, integrating elements of dignity therapy and music medicine by actively engaging patients and families in care. However, given the absence of a control group and independent assessors, these results should be interpreted with caution, and future studies incorporating controlled designs are needed to more rigorously evaluate outcomes and replicability.

A recent systematic review of music interventions in palliative care found that studies lacked sufficient methodological detail to ensure replicability, thereby limiting meta-analytic synthesis and broader clinical recommendations (Pérez-Eizaguirre and Vergara-Moragues 2021). Current evidence in music medicine has also been characterized by a high risk of bias and low certainty of findings (Bradt et al. 2015; Ambler et al. 2021), indicating a need for more rigorous study designs. This priority aligns with the goals of the National Institutes of Health’s Sound Health Initiative (Sound Health: An NIH-Kennedy Center Partnership n.d.) and informed the development of the Musical Rounds training and facilitator guide, which are both available upon request.

There is some literature regarding potential harm in music medicine and music therapy (Silverman et al. 2020; Murakami 2021). In some cases, it appears that Musical Rounds offered a space for participants to bring buried thoughts and feelings to the surface, sometimes revealing increased pain, stress, and anxiety surrounding existential concerns and anticipatory grief (Coelho and Barbosa 2017; Johnson et al. 2017). Symptoms of anticipatory grief, however, are a natural part of the coping process and worsening in a reported outcome may not always be detrimental in this context. It does, however, highlight the importance of a close relationship with clinical teams for debriefing with a participant in the case of a particularly emotional session.

Referrals for Musical Rounds were made directly by palliative care and ICU teams to facilitate collaboration with the clinical care teams. Given the sensitive context of palliative care, this initial referral process was considered necessary to ensure appropriateness, as some situations required discretion before engagement (a patient’s final moments, isolation precautions, interpersonal conflict). It is notable that not all hospitalized patients with life-limiting illnesses were followed by palliative care, suggesting that future implementation could benefit from additional referral pathways to increase reach and inclusivity.

One musician-clinician performed all Musical Rounds sessions, from consent through post-session survey. The survey responses were collected by the same individual providing the session. This introduces a risk of reporting bias, which we aimed to mitigate by performing a chart review to compare pain reported to nursing versus the musician-clinician. In a future study, however, having a separate individual for data collection in addition to the musician would eliminate this potential for bias.

This study was not a randomized controlled trial, and therefore causal inferences cannot be made. Future research should examine whether the combined use of interview and personalized music offers distinct benefits compared to either component alone. An active control condition (such as a therapeutic conversation involving the same questions without music) could help isolate potential mechanisms. Comparisons with existing data on dignity therapy interventions may also provide useful context for interpreting outcomes.

Regarding outcome measures, the NRS provided a quick, reliable, and validated approach to assess pain, stress, anxiety, and comfort. However, some responses demonstrated ceiling or floor effects, with values clustering toward the scale’s extremes. Incorporating additional measures, such as more comprehensive psychological assessments and biometric indicators (e.g. vital signs, electroencephalogram {EEG}, or vascular endothelial function), could provide additional insight into the effects of the sessions. Gathering staff perspectives would further enrich understanding of the intervention’s perceived impact and feasibility.

Despite these limitations, this study contributes to a limited but growing body of research exploring patient- and family-centered music interventions in palliative care. The findings highlight the potential of Musical Rounds as a feasible and replicable approach deserving of further controlled investigation.

### What this study adds

The goal of Musical Rounds is to cultivate human connection through live music and conversation for those with serious illness, enhancing dignity, connection with loved ones, and inspiring humanistic care. We hope these findings highlight that music medicine sessions such as Musical Rounds have the potential to positively impact not only patients but also their loved ones.

## Supporting information

10.1017/S1478951526102624.sm001 Ambler et al. supplementary material 1 Ambler et al. supplementary material

10.1017/S1478951526102624.sm002 Ambler et al. supplementary material 2 Ambler et al. supplementary material

10.1017/S1478951526102624.sm003 Ambler et al. supplementary material 3 Ambler et al. supplementary material

## Data Availability

Data, facilitator guide, and study protocol are stored on an encrypted server with the corresponding author (MA) and are available from the corresponding author on reasonable request.

## Supplementary material

The supplementary material for this article can be found at https://doi.org/10.1017/S1478951526102624.

## Figure 1 Long description

The infographic illustrates a four-step process. Step 1, labeled 'Referrals', involves palliative or ICU rounds, meeting the patient or loved one and obtaining written consent for participation. Step 2, 'Musician researcher facilitates', includes a pre-survey, recording of conversation and improvised music and a post-survey. Step 3, 'Post-Production', involves editing the recording for interruptions and clarity and layering music underneath the story. Step 4, 'Follow-up', includes sending the participant a recording via email and text and an optional satisfaction or feedback survey with the recording.

Navigate back to Figure 1.

## Figure 2 Long description

It splits into two main categories: 'Consented' with 100 individuals and 'Declined to participate' with 56 individuals. Under 'Consented', 54 were patients alone, 36 were patients with a loved one and 16 were loved ones alone. 'Complete Live Session' shows 99 patients taken to procedure, with one interruption. 'Lost to follow-up' includes 16 who did not respond after three contact attempts. 'Complete feedback survey' notes 41 acknowledged receipt of recordings, with 43 not completing the survey. 'Declined to participate' reasons include symptom burden (11), requested another time (11), not interested (11), too tired (10), requested music only (7) and other reasons (2).

Navigate back to Figure 2.

## Figure 3 Long description

The image contains two boxplots comparing pre and post numerical rating scale outcomes. The y-axis is labeled 'Numerical Rating Scale' ranging from 0 to 10. Asterisks above the boxes indicate significant differences, with three asterisks denoting p less than .001. The bottom boxplot represents family member data, with similar categories: Family Pre Pain, Family Post Pain, Family Pre Stress, Family Post Stress, Family Pre Anxiety, Family Post Anxiety, Family Pre Comfort and Family Post Comfort. The y-axis is similarly labeled 'Numerical Rating Scale' ranging from 0 to 10. Asterisks indicate significant differences, with three asterisks denoting p less than .001 and two asterisks denoting p less than .01.

Navigate back to Figure 3.

## Table 1 Long description

The table presents demographic and characteristic data for 100 patients and 48 family members. Patients are primarily from Stanford Hospital Center (59%), with a mean age of 63.31 years and a gender distribution of 57 females to 43 males. Family members, with a gender ratio of 31 females to 17 males, are mostly spouses (40%) and children (29%). The majority of patients are White (44%) and non-Hispanic (72%), with English as the predominant language (96%). Oncology is the most common diagnosis (70%), and 89% of patients were referred by Palliative Care. Sessions were mostly conducted with patients alone (51%), and 74% consented to public sharing of recordings. The median length of stay prior to sessions was 10 days, with a wide range from 1 to 133 days.

Navigate back to Table 1.

## Table 2 Long description

The table compares pre- and post-session numerical ratings for pain, stress, anxiety, and comfort among patients and their loved ones. Significant reductions in stress and anxiety were observed for both groups, with p-values <.001. Comfort levels increased significantly post-session, with p-values <.001 for patients and 0.004 for loved ones. Pain ratings decreased significantly for patients (p<.001) but not for loved ones (p=0.352). The sample sizes varied, particularly for comfort, due to survey modifications during recruitment. These results suggest the intervention effectively reduced stress and anxiety while improving comfort, with mixed results for pain relief.

Navigate back to Table 2.
