# Supplementary material for: Musical Rounds: A multisite, mixed-methods feasibility study of a musical legacy session in palliative care
Source: Palliat Support Care. 2026 May 7;24:e135. doi: 10.1017/S1478951526102624 (PMC13202410; doi:10.1017/S1478951526102624)
Supplement: Ambler et al. supplementary material 3 — Ambler et al. supplementary material [file S1478951526102624sup003.pdf]

Patient demographics

Patient ID	______________________ (initials_date (MA_052124))
MRN	______________________
Name	______________________
Gender	◯ M<br>◯ F<br>◯ non-binary
Race	______________________
Ethnicity	______________________
Language spoken	______________________
DOB	______________________
Date of admission	______________________
Date of intervention	______________________
Location	◯ SHC<br>◯ PAVA<br>◯ SCVMC<br>◯ other
Service	☐ Palliative care<br>☐ Hospice<br>☐ Spiritual care<br>☐ Oncology<br>☐ Other
Comfort Care?	◯ Yes<br>◯ No
Intervention start time	______________________
Diagnosis (as indicated in HPI)	______________________

Contact information

Who is participating?	◯ Patient alone<br>◯ Family alone<br>◯ Patient and family together
How many loved ones are in the room?	__________________________
Palliative care team member in room?	◯ Yes<br>◯ No
Patient name	__________________________
Patient email	__________________________
Patient phone	__________________________
Family name	__________________________
Relationship to patient	__________________________
Family gender	◯ Male<br>◯ Female<br>◯ Non-binary<br>◯ Prefer not to answer
Family age	__________________________
Family email	__________________________
Family phone	__________________________

Pre-intervention questions

How would you rate your pain right now?	◯ 0<br>◯ 1<br>◯ 2<br>◯ 3<br>◯ 4<br>◯ 5<br>◯ 6<br>◯ 7<br>◯ 8<br>◯ 9<br>◯ 10
Family: How would you rate your pain right now?	◯ 0<br>◯ 1<br>◯ 2<br>◯ 3<br>◯ 4<br>◯ 5<br>◯ 6<br>◯ 7<br>◯ 8<br>◯ 9<br>◯ 10
How would you rate your level of stress right now?	◯ 0<br>◯ 1<br>◯ 2<br>◯ 3<br>◯ 4<br>◯ 5<br>◯ 6<br>◯ 7<br>◯ 8<br>◯ 9<br>◯ 10
Family: How would you rate your level of stress right now?	◯ 0<br>◯ 1<br>◯ 2<br>◯ 3<br>◯ 4<br>◯ 5<br>◯ 6<br>◯ 7<br>◯ 8<br>◯ 9<br>◯ 10

How would you rate your level of anxiety right now?	◯ 0<br>◯ 1<br>◯ 2<br>◯ 3<br>◯ 4<br>◯ 5<br>◯ 6<br>◯ 7<br>◯ 8<br>◯ 9<br>◯ 10
Family: How would you rate your level of anxiety right now?	◯ 0<br>◯ 1<br>◯ 2<br>◯ 3<br>◯ 4<br>◯ 5<br>◯ 6<br>◯ 7<br>◯ 8<br>◯ 9<br>◯ 10
How much do you think music-based activities can help in caring for hospitalised patients? 0=not at all helpful to 10=extremely helpful.	◯ 0<br>◯ 1<br>◯ 2<br>◯ 3<br>◯ 4<br>◯ 5<br>◯ 6<br>◯ 7<br>◯ 8<br>◯ 9<br>◯ 10
Family: How much do you think music-based activities can help in caring for hospitalised patients? 0=not at all helpful to 10=extremely helpful.	◯ 0<br>◯ 1<br>◯ 2<br>◯ 3<br>◯ 4<br>◯ 5<br>◯ 6<br>◯ 7<br>◯ 8<br>◯ 9<br>◯ 10
Who should be offered music based activities in hospital?	__________________________________
Family: Who should be offered music based activities in hospital?	__________________________________

How would you rate your level of comfort right now?	◯ 0<br>◯ 1<br>◯ 2<br>◯ 3<br>◯ 4<br>◯ 5<br>◯ 6<br>◯ 7<br>◯ 8<br>◯ 9<br>◯ 10
Family: How would you rate your level of comfort right now?	◯ 0<br>◯ 1<br>◯ 2<br>◯ 3<br>◯ 4<br>◯ 5<br>◯ 6<br>◯ 7<br>◯ 8<br>◯ 9<br>◯ 10
Patient: How would you describe your mood right now?	__________________________________
Family: how would you describe your mood right now?	__________________________________
Why did you choose to participate in this project?	__________________________________
Family: why did you choose to participate in this project?	__________________________________
What role has music played in your life?	__________________________________
Family: What role has music played in your life?	__________________________________
Favorite song? Artists? Instrument? Genre?	__________________________________

Intervention

Which questions were asked	☐ Describe a time you felt overwhelming joy<br>☐ How did you meet your partner?<br>☐ Transport me to your favorite place on Earth<br>☐ Describe a favorite meal. Who is with you? Where are you?<br>☐ Tell me a story from your childhood.<br>☐ Think about the people who know you best. What adjectives would they use to describe you?<br>☐ Other
If other, what did you ask?	__________________________________

Post-intervention

End time ______________________

How would you rate your pain right now?
- ◯ 0
- ◯ 1
- ◯ 2
- ◯ 3
- ◯ 4
- ◯ 5
- ◯ 6
- ◯ 7
- ◯ 8
- ◯ 9
- ◯ 10

Family: how would you rate your pain right now?
- ◯ 0
- ◯ 1
- ◯ 2
- ◯ 3
- ◯ 4
- ◯ 5
- ◯ 6
- ◯ 7
- ◯ 8
- ◯ 9
- ◯ 10

How would you rate your level of stress right now?
- ◯ 0
- ◯ 1
- ◯ 2
- ◯ 3
- ◯ 4
- ◯ 5
- ◯ 6
- ◯ 7
- ◯ 8
- ◯ 9
- ◯ 10

Family: How would you rate your level of stress right now?
- ◯ 0
- ◯ 1
- ◯ 2
- ◯ 3
- ◯ 4
- ◯ 5
- ◯ 6
- ◯ 7
- ◯ 8
- ◯ 9
- ◯ 10

How would you rate your level of anxiety right now?	◯ 0<br>◯ 1<br>◯ 2<br>◯ 3<br>◯ 4<br>◯ 5<br>◯ 6<br>◯ 7<br>◯ 8<br>◯ 9<br>◯ 10
Family: How would you rate your level of anxiety right now?	◯ 0<br>◯ 1<br>◯ 2<br>◯ 3<br>◯ 4<br>◯ 5<br>◯ 6<br>◯ 7<br>◯ 8<br>◯ 9<br>◯ 10
How much do you think music-based activities can help in caring for hospitalised patients? 0=not at all helpful to 10=extremely helpful.	◯ 0<br>◯ 1<br>◯ 2<br>◯ 3<br>◯ 4<br>◯ 5<br>◯ 6<br>◯ 7<br>◯ 8<br>◯ 9<br>◯ 10
Family: How much do you think music-based activities can help in caring for hospitalised patients? 0=not at all helpful to 10=extremely helpful.	◯ 0<br>◯ 1<br>◯ 2<br>◯ 3<br>◯ 4<br>◯ 5<br>◯ 6<br>◯ 7<br>◯ 8<br>◯ 9<br>◯ 10
How often do you get to have conversations like this where you talk about life and things meaningful for you?	◯ never<br>◯ annually<br>◯ monthly<br>◯ weekly<br>◯ daily
Family: How often do you get to have conversations like this where you talk about life and things meaningful for you?	◯ never<br>◯ annually<br>◯ monthly<br>◯ weekly<br>◯ daily

How would you rate your level of comfort right now?	◯ 0<br>◯ 1<br>◯ 2<br>◯ 3<br>◯ 4<br>◯ 5<br>◯ 6<br>◯ 7<br>◯ 8<br>◯ 9<br>◯ 10
Family: How would you rate your level of comfort right now?	◯ 0<br>◯ 1<br>◯ 2<br>◯ 3<br>◯ 4<br>◯ 5<br>◯ 6<br>◯ 7<br>◯ 8<br>◯ 9<br>◯ 10
Patient: How would you describe your mood right now?	__________________________________
Family: how would you describe your mood right now?	__________________________________
What other feedback do you have for us that you particularly enjoyed or feel could have been improved?	__________________________________
Consent to share recordings	◯ Yes to just patient<br>◯ Yes to patient and family<br>◯ Yes to public<br>◯ No
Raw recording length (min)	____________________________
Final mixed recording length (min)	____________________________

Call/email feedback

Hello,

Thank you so much for being a part of the Musical Rounds project and sharing you or your family member's story with us. We truly hope you enjoy the recordings we provided with you.

As a last effort to improve our own project, we would greatly appreciate if you could complete the survey below. It will help us understand what feedback you may have for us.

Thank you so much! If you have any questions or concerns, please email Melanie Ambler at mambler@stanford.edu

Warmly,

Musical Rounds Research Team

---

68) Patient: how satisfied are you with Musical Rounds?

- ☐ Very satisfied
- ☐ Satisfied
- ☐ Neutral
- ☐ Dissatisfied
- ☐ Very dissatisfied

---

69) Family: how satisfied are you with Musical Rounds?

- ☐ Very satisfied
- ☐ Satisfied
- ☐ Neutral
- ☐ Dissatisfied
- ☐ Very dissatisfied

---

70) Do you have any suggestions to improve the project for future patients and families?

____

---

71) What worked particularly well for you throughout this Musical Rounds experience?

____

---

72) After hearing the recording of your story and soundtrack, would you be willing for this recording to be shared with the public?

- ◯ Yes, you can share both my story and the music
- ◯ Yes, but only the music can be shared
- ◯ Yes, but only the story can be shared
- ◯ No, I would like this kept private

---

73) If you feel comfortable sharing your story/music publicly, why did you say yes?

____
